# One-step generation of multiple transgenic mouse lines using an *i*mproved Pronuclear Injection-based Targeted Transgenesis (*i*-PITT)

**DOI:** 10.1186/s12864-015-1432-5

**Published:** 2015-04-09

**Authors:** Masato Ohtsuka, Hiromi Miura, Keiji Mochida, Michiko Hirose, Ayumi Hasegawa, Atsuo Ogura, Ryuta Mizutani, Minoru Kimura, Ayako Isotani, Masahito Ikawa, Masahiro Sato, Channabasavaiah B Gurumurthy

**Affiliations:** Department of Molecular Life Science, Division of Basic Medical Science and Molecular Medicine, Tokai University School of Medicine, 143 Shimokasuya, Isehara, Kanagawa 259-1193 Japan; RIKEN BioResource Center, 3-1-1 Koyadai, Tsukuba, Ibaraki 305-0074 Japan; Graduate School of Life and Environmental Science, University of Tsukuba, 1-1-1 Ten-noudai, Tsukuba, Ibaraki 305-8572 Japan; Graduate School of Engineering, Tokai University, Kitakaname, Hiratsuka, Kanagawa 259-1292 Japan; Immunology Frontier Research Center, Osaka University, 3-1 Yamadaoka, Suita, Osaka 565-0871 Japan; Research Institute for Microbial Diseases, Osaka University, 3-1 Yamadaoka, Suita, Osaka 565-0871 Japan; Section of Gene Expression Regulation, Frontier Science Research Center, Kagoshima University, 8-35-1 Sakuragaoka, Kagoshima, Kagoshima 890-8544 Japan; Mouse Genome Engineering Core Facility, Department of Genetics, Cell Biology and Anatomy, University of Nebraska Medical Center, Omaha, NE 68198 USA

**Keywords:** Transgenic mouse, Pronuclear injection-based targeted transgenesis, Cre-*lox*P, PhiC31-*attP*/*B*, FLP-*FRT*, *Rosa26*, Transportation of the cauda epididymides

## Abstract

**Background:**

The pronuclear injection (PI) is the simplest and widely used method to generate transgenic (Tg) mice. Unfortunately, PI-based Tg mice show uncertain transgene expression due to random transgene insertion in the genome, usually with multiple copies. Thus, typically at least three or more Tg lines are produced by injecting over 200 zygotes and the best line/s among them are selected through laborious screening steps. Recently, we developed technologies using Cre-*lox*P system that allow targeted insertion of single-copy transgene into a predetermined locus through PI. We termed the method as PI-based Targeted Transgenesis (PITT). A similar method using PhiC31-*attP*/*B* system was reported subsequently.

**Results:**

Here, we developed an *i*mproved-PITT (*i*-PITT) method by combining Cre-*lox*P, PhiC31-*attP*/*B* and FLP-*FRT* systems directly under C57BL/6N inbred strain, unlike the mixed strain used in previous reports. The targeted Tg efficiency in the *i*-PITT typically ranged from 10 to 30%, with 47 and 62% in two of the sessions, which is by-far the best Tg rate reported. Furthermore, the system could generate multiple Tg mice simultaneously. We demonstrate that injection of up to three different Tg cassettes in a single injection session into as less as 181 zygotes resulted in production of all three separate Tg DNA containing targeted Tg mice.

**Conclusions:**

The *i*-PITT system offers several advantages compared to previous methods: multiplexing capability (*i*-PITT is the only targeted-transgenic method that is proven to generate multiple different transgenic lines simultaneously), very high efficiency of targeted-transgenesis (up to 62%), significantly reduces animal numbers in mouse-transgenesis and the system is developed under C57BL/6N strain, the most commonly used pure genetic background. Further, the *i*-PITT system is freely accessible to scientific community.

**Electronic supplementary material:**

The online version of this article (doi:10.1186/s12864-015-1432-5) contains supplementary material, which is available to authorized users.

## Background

Transgenic (Tg) mice have been extensively used for *in vivo* analysis of gene function and generation of human disease models. Since generation of the first Tg mice was reported by Gordon et al. in 1980, who performed pronuclear injection (PI) of purified DNA into zygotes, PI-mediated transgenesis has been the most common method for more than 30 years [1-3]. In general, for obtaining several Tg mouse lines harboring a specific transgene, it typically requires approximately 200 or more eggs. Unfortunately, the PI-mediated transgenesis is often associated with wide variability in the level and pattern of transgene expression, which causes phenotypic variability among individual Tg lines, probably due to the random nature of copy number, configuration, and insertion site of the transgene. Due to such variability in transgene expression, researchers need to extensively screen various lines and choose the most suitable Tg mouse line(s) showing desired transgene expression, which is a very laborious and time-consuming task [4].

To overcome the pitfalls associated with random transgenesis, single-copy insertion of a transgene into a predetermined genomic locus can be accomplished using Embryonic Stem (ES) cell-mediated gene targeting, which is the gold-standard for generation of targeted Tg mice [4]. However, this approach is laborious, expensive, and time-consuming when compared to PI-based transgenesis. Strategies using recombinase- or integrase-based targeted insertion through PI have been recently described by us and others [4-7]. We termed this technology as “Pronuclear Injection-based Targeted Transgenesis (PITT). The PITT system requires a ‘seed mice strain’ containing a suitable landing pad at a predetermined genomic locus as an embryo donor for PI. The method requires, in its initial stage, to establish the seed mouse by targeted insertion of the landing pad via conventional ES cell-based gene targeting approach. Such an established mouse line serves as a “seed” strain, from which Tg mice harboring the DNA of interest (DOI) are generated by injection of donor vector into zygotes along with DNA/mRNA for recombinase or integrase. For performing targeted insertion of DOI using a seed strain, we and another group used Cre-*lox*P recombination system [6,7], while a third group used PhiC31 integrase system [5].

Despite the advantages of PITT approach compared to traditional methods, its full potential is not discovered yet, because the available methods are only single-enzyme based PITT versions. We hypothesized that further improving of the PITT by combining multiple recombinase-integrase systems, the method can be made more efficient, versatile and capable of multiplexing. Specifically, we anticipated that such a strategy should make the PITT method able to rapidly generate multiple different transgenic lines simultaneously, that are targeted precisely to the same genomic location. This would ultimately facilitate many areas of biomedical research that depend on rapid generation of multiple, reliable transgenic mice since it would address the major constraints such as time, costs and variable efficiencies associated with the use of ES cell-based targeted transgenesis approach while taking care of several pitfalls associated with random transgenesis approaches. Therefore, in this report we further improved the technique by creating the next generation seed mouse with a combination of elements including mutant *lox*P, mutant *FRT*, and *attP* sites at the *Rosa26* locus. We termed the new seed mouse as TOKMO-3 (*Tok*ai *M*asato *O*htsuka no.*3*) and the method as *i*mproved*-*PITT (*i-*PITT). This system adds many new features and capabilities to the previously described PITT, and to the targeted transgenesis approaches in general. These features are: 1) The *i-*PITT method enables the use of any of the three insertion systems, viz. Cre, PhiC31 or FLP, or combinations, for inserting the Tg cassettes. 2) Simultaneous use of two systems, for e.g., Cre-*lox*P-mediated recombination and PhiC31 integration, significantly enhances the targeted insertion efficiency. 3) Many Tg DOI constructs can be co-injected to produce Tg lines for each of the constructs injected. 4) The multiplexing capability allows the generation of transgenic lines with 70 zygotes per donor Tg construct or less (three separate DOI containing Tg lines were produced in an injection session using less than 200 zygotes). 5) The new seed mouse is under the C57BL/6N genetic background, which is widely used as a standard strain by the scientific community, including the International Knockout Mouse Consortium (IKMC) [8]. Recently there have been remarkable advances in knockout technology using CRISPR/Cas9 system in which multiple gene knockouts can be made in a single microinjection session. The *i-*PITT method described here offers the advantage of creating multiple Tg models in a single experimental session and thus serves as an analogous tool for multiplexing in Tg technology similar to CRISPR/Cas9 tool for KO technology.

## Results

### *i-*PITT Strategy

**Figure 1:**
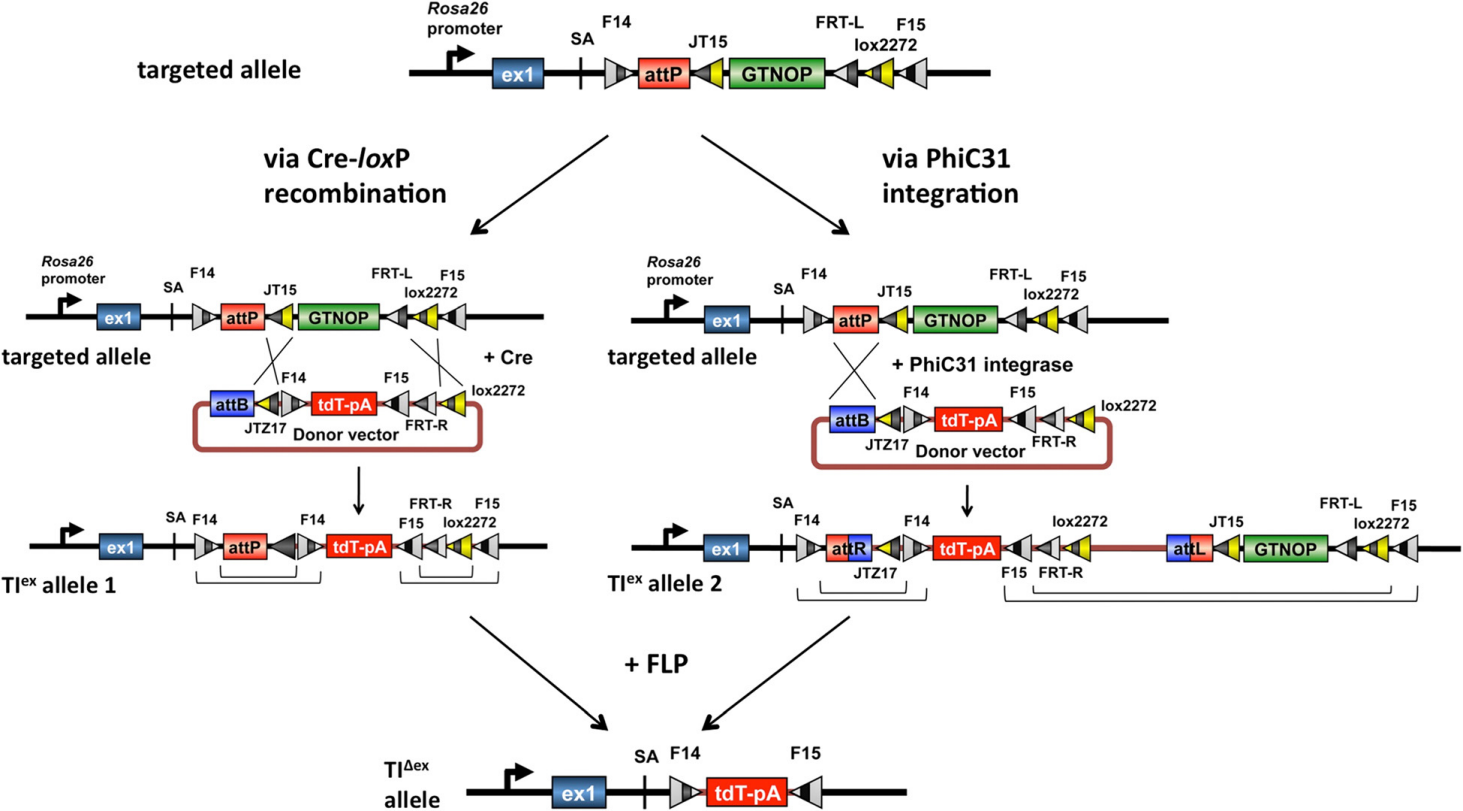
**Schematic of PITT-mediated targeted insertion using the new seed mice, TOKOM-3.** Cre-PITT (via mutant *lox*P sites) and PhiC31-PITT (via *attB*/*P* site) confer insertion of the donor vector containing. DNA Of Interest (DOI), into a target site in the genome, to generate TI^ex^ allele 1 or 2 respectively. DOI shown in this example is tdTomato-poly(A) cassette in this figure. Introduction of FLP removes extra sequence in the TI^ex^ allele 1 and 4, resulting in generation of TI^Δex^ allele. See Additional file 1: Figures S1, S3 and S4 for other possible outcomes recombination. GTNOP: a cassette containing “e*G*FP-*T*2A-*N*eomycin resistant gene-h*O*CT4-*P*olyA”.

The overall scheme of *i-*PITT is illustrated in Figure 1. A new PITT seed mouse (TOKMO-3) under C57BL/6N genetic background was engineered by inserting a landing pad containing *lox*P derivatives (*JT15* and *lox2272*), *attP*, and *FRT* derivatives (*F14*, *F15* and *FRT-L*) into the *Rosa26* locus through ES cell-based targeting. A donor vector carrying tdTomato-pA, *lox*P derivatives (*JTZ17* and *lox2272*), *attB*, and *FRT* derivatives (*F14*, *F15* and *FRT-R*) was designed to perform site-specific insertion of a transgene (e.g. “tdTomato-pA” in Figure 1). The transgene flanked by the *F14* and *F15* sites in the donor vector was inserted into target locus of fertilized eggs via *i-*PITT (Figure 1 and Additional file 1: Figure S1) that offers options for any of the three enzymes Cre, PhiC31 or FLP to choose for targeted insertion. The resulting embryos will exhibit “targeted insertion with extra sequence (TI^ex^)” allele. The strategy is designed in such way that the extra sequences will be flanked by *F14* and *F15* elements which enable the removal of extra sequences by breeding with FLP mouse. This results in cleanly inserted transgene flanked by only the *F14* and *F15* sites (“TI^Δex^” allele). Although the structures of TI^ex^ allele differ among the site-specific insertion systems employed (e.g. TI^ex^ allele 1 and 2; Figure 1), the structure of final TI^Δex^ alleles generated will be identical in all the strategies. Combination of mutant *lox*Ps (*JT15*/*JTZ17* and *lox2272*) was same as in our original PITT system [6]. Among the multitude of mutant *FRT* sites reported previously, we chose *F14* and *F15* (the spacer variants) and *FRT-L*/*R* (inverted repeat variant), since *F14*/*F15* pairing was known to be incompatible [9] and thus we anticipated that combination of the spacer variants and the inverted repeat variant would show high recombination efficiency similar to *JT15*/*JTZ17* and *lox2272* combinations for the Cre-*lox*P system [6]. The targeted allele also contains a 43-bp fragment corresponding to human OCT4 gene sequence [described in Hockemeyer et al. [10]] that can serve as a zinc finger nuclease target to aid in insertion of additional sequences for future needs, but the component is not tested yet.

### Verification of landing pad insertion in ES cells

**Figure 2:**
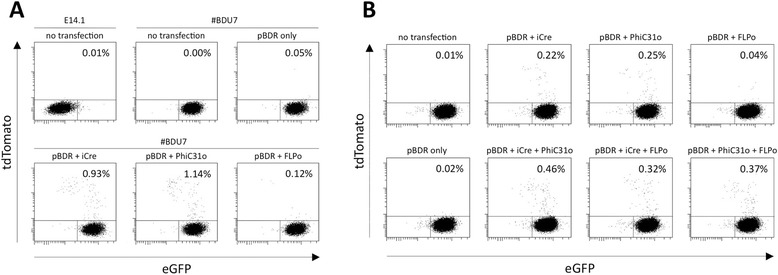
**Targeted insertion in ES cells. (A)** Introduction of donor vector (pBDR) harboring promoter-less tdTomato gene into targeted ES cells (#BDU7) with each expression vector for iCre recombinase, FLPo recombinase or PhiC31 integrase. Red fluorescent ES cells, as analyzed by FACS, are regarded as successfully targeted tdTomato cassette inserted cells. **(B)** Co-introduction of combinations of recombinases or integrase expression vectors increases transgene insertion efficiency.

To confirm that three kinds of landing pads work in the genomic context, we first established an ES cell clone (termed #BDU7) that contains mutant *lox*P, mutant *FRT* and *attP* sites at the *Rosa26* locus. The #BDU7 was then transfected with a pBDR donor vector carrying a promoter-less tdTomato gene, the recombinase (iCre and/or FLPo) and/or integrase (PhiC31o) expression vectors. The tdTomato expression will be driven by endogenous *Rosa26* promoter only if a correct insertion of the cassette at the target locus occurs. The red fluorescence in successfully targeted cells was detected by FACS analysis. Two days after transfection, approximately 1% of cells were positive for tdTomato fluorescence when iCre- or PhiC31o-expression vector (5 μg per experiment) was used (Figure 2A), but when FLPo-expression vector was used, only a few cells showed tdTomato fluorescence (Figure 2A). Notably, the percentage of fluorescent cells increased when any two expression vectors (iCre and PhiC31o or iCre and FLPo or PhiC31o and FLPo expression vectors; all 2.5 μg each) were co-transfected (Figure 2B and Additional file 1: Figure S2B). We then tested if inclusion of six copies of tandem repeats of recombination/integration sites in the donor vector would increase the insertion efficiency (Additional file 1: Figure S2A and S2B) and found that it was slightly improved (0.90% to 1.21%) in the Cre-*lox*P-based system, when 6x*JTZ17* was included in a donor vector. No obvious improvement was noticed in the PhiC31-based system (Additional file 1: Figure S2B).

These results indicated that 1) the landing pad architecture we designed worked as expected, and 2) the Cre-based and PhiC31-based gene insertion systems showed comparable targeted insertion efficiencies, while overall efficiency of the FLP-based system was relatively poor compared to PhiC31- and Cre-based systems, 3) the combinatorial use of two insertion systems (Cre- and PhiC31-based) increased the targeted insertion efficiency, and 4) tandem repeats of recombinase recognition sites in the donor vectors enhanced Cre-*lox*P-mediated targeted insertion of DOI.

### Validation of *i-*PITT

After confirming that desired DNA cassettes can be successfully inserted into the landing pads by using recombinases and/or integrase in ES cells, a novel seed mouse line was established using C57BL/6N-derived ES cell clones harboring the targeted allele. Similar to our previous seed mouse (TOKMO-1: *Rosa26* seed mouse with mixed genetic background of C57BL/6J and 129/Ola), both homozygotes and heterozygotes of the new seed mouse (termed TOKMO-3, line BDT#73) were viable with normal reproductive ability.

To test PhiC31-based PITT on the new seed mouse TOKMO-3, we injected various concentrations of PhiC31o mRNA along with 10 ng/μl of pBER donor vector (similar to pBDR, without the hygromycin expression cassette) into fertilized eggs that were generated by *in vitro* fertilization (IVF) of C57BL/6N eggs with the epididymal spermatozoa isolated from a male homozygous TOKMO-3 mouse. Based on the results (survival rates and targeting efficiency) presented in Additional file 1: Table S1, we decided to use 7.5 or 15 ng/μl of PhiC31o mRNA in subsequent experiments. These concentrations are relatively higher compared to iCre mRNA concentrations (0.5 or 1.0 ng/μl) that is typically needed for PITT [11].

**Figure 3:**
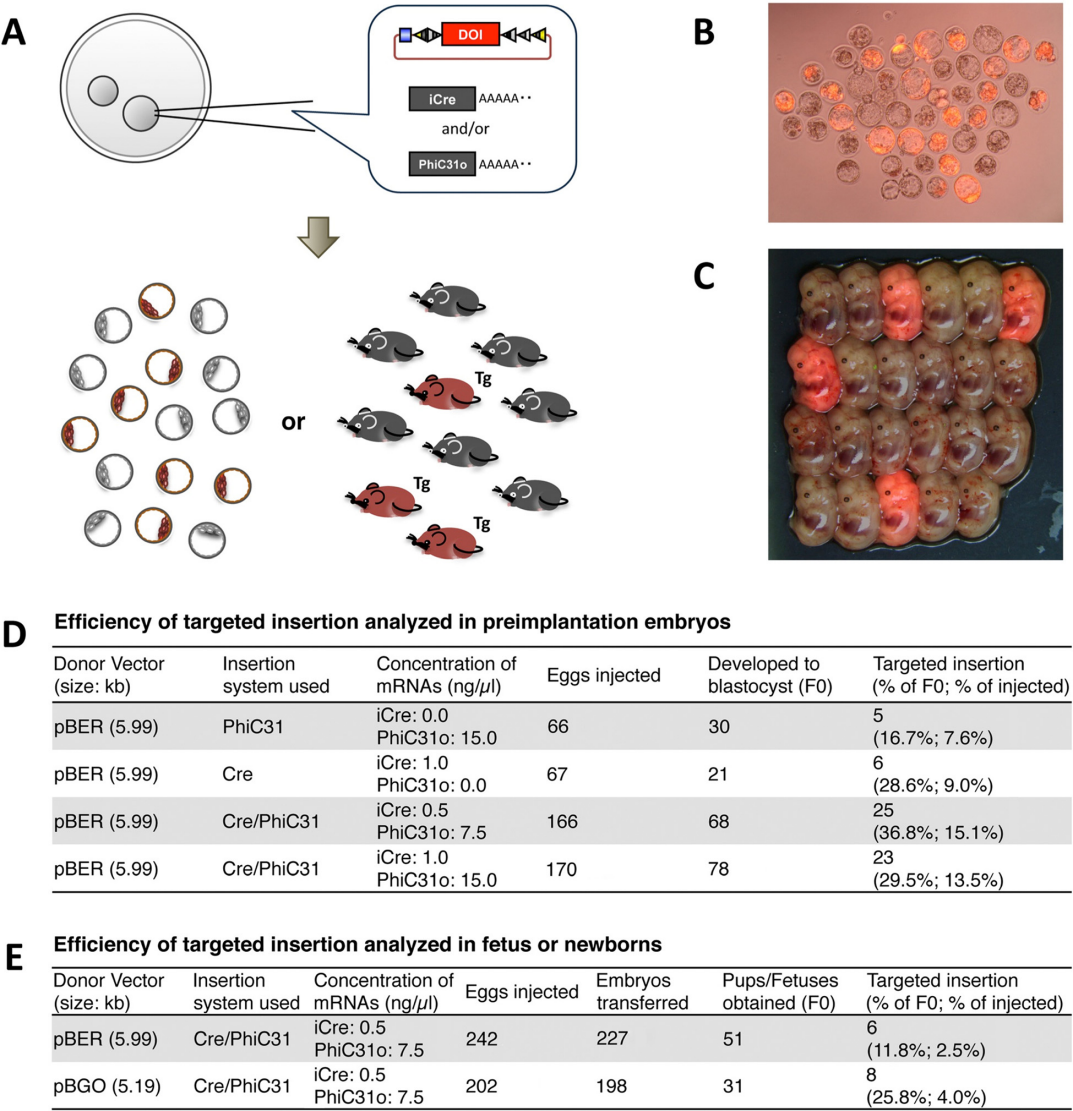
**PITT using TOKOM-3 seed mice. (A)** Schematic diagram of the PITT injection experiment. Donor vector containing DOI is injected into the fertilized eggs harboring landing pad together with iCre and/or PhiC31o mRNA. **(B)** Successful targeted transgenesis in blastocysts derived from zygotes injected with pBER + iCre mRNA + PhiC31o mRNA. **(C)** Successful targeted transgenesis in day 13.5 fetuses. Zygotes/fetuses exhibiting red fluorescence indicate successful targeted insertion of DOI. **(D, E)** The PITT results in blastocysts **(D)** and fetuses/pups **(E)**.

The PITT efficiency for Cre- or PhiC31-based systems was assessed by injecting zygotes, culturing them until blastocysts, and subsequent analysis for targeted insertion (Figure 3A and B). Targeted insertion was detected in 28.6% or 16.7% of developed blastocysts (9.0% or 7.6% of total injected zygotes) when iCre mRNA or PhiC31o mRNA were injected (Figure 3D). Interestingly, co-injection of iCre and PhiC31o mRNAs resulted in increased insertion efficiency of up to 36.8% of developed blastocysts (up to 15.1% of total injected zygotes) (Figure 3D). Notably, insertion rate as high as 61.5% among developed blastocysts was achieved in a single experiment (Experiment 7 in Additional file 1: Table S2). Based on these results, we decided to follow a co-injection strategy using iCre mRNA and PhiC31o mRNA at concentrations 0.5 ng/μl and 7.5 ng/μl respectively in subsequent experiments.

We next examined whether co-injection of iCre and PhiC31o mRNAs into zygotes leads to production of targeted Tg fetuses or newborns (Figure 3A and C). Two different donor vectors were included in the injection, pBER and pBGO. The targeted insertion efficiencies were 11.8% (for fetuses) and 25.8% (for newborns), and 2.5% and 4.0% for total injected zygotes, respectively (Figure 3E).

These results indicate that 1) both PhiC31 and iCre-based targeted insertions were effective in using the new seed mouse TOKMO-3, and that 2) combinatorial use of the two systems enhanced the *in vivo* targeted insertion efficiency.

### Simultaneous production of multiple different targeted Tg mouse lines using *i-*PITT

Given the ability of the original PITT system to insert a single copy transgene through specially designed donor vectors, we hypothesized that when a mixture of multiple donor vectors is injected into zygotes, the resulting independent Tg founder mouse lines should contain only one type of transgene from the mixture. If this occurs, more than two types of Tg lines can be produced at once in a single injection event, which offers versatility to the system and also would reduce the cost significantly, compared to the traditional Tg production method.

**Figure 4:**
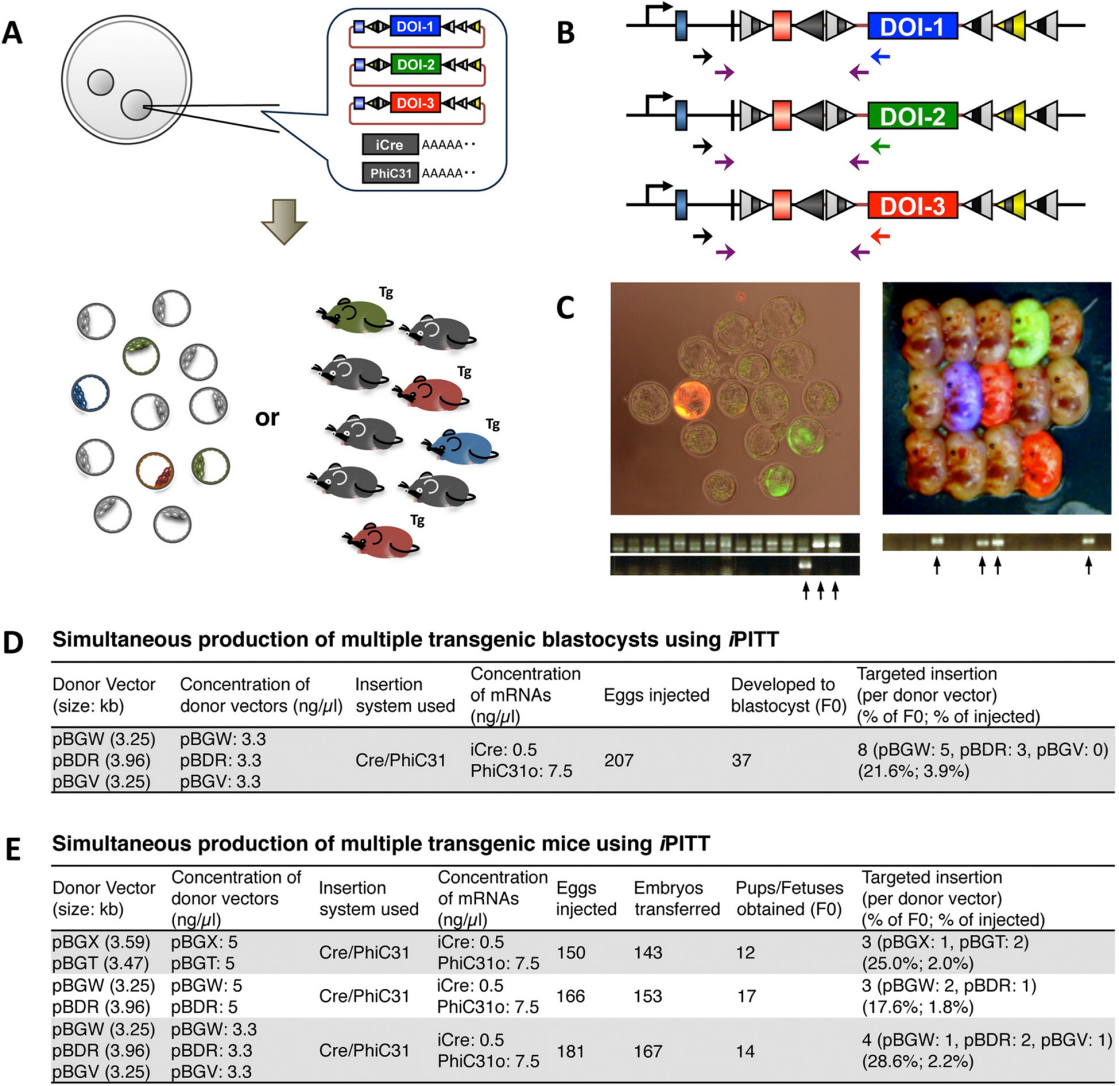
**Production of multiple targeted Tg mouse lines using** ***i-*** **PITT. (A)** Schematic of simultaneous production of multiple Tg lines using *i-*PITT. Multiple donor vectors that harbor different DOI are mixed and co-injected with iCre and PhiC31o mRNA into the fertilized eggs carrying the *i-*PITT landing pad in their genome. Appearance of different fluorescent colors indicates successful insertion of DOI. **(B)** Schematic of targeted insertion alleles for each DOI. TI^ex^ allele 1 is shown as an example. Arrows indicate the PCR primer sets used for genotype identification of the correct insertion. For detecting targeted transgenesis in blastocyst, 1st PCR was performed using the outer most primer pair sets (black and blue or black and green or black and red arrows) and nested PCR using the internal primer pair sets (purple arrows). For detecting targeted transgenesis in fetuses, PCR with only the purple primer pair is sufficient. **(C)** Example of simultaneous production of multiple targeted Tgs. Blastocysts (left panel) and day 13.5 fetuses (right panel) derived from injected zygotes. Zygotes/fetuses exhibiting blue, green or red fluorescence indicate successful insertion of DOI from pBGV, pBGW or pBDR vectors respectively. The results of PCR-based genotyping are shown below the images; arrows indicate positive samples. **(D, E)** The results of *i-*PITT experiment in blastocyst embryos **(D)** and fetuses/pups **(E)**.

As a preliminary test, we first assessed this possibility by injecting a mixture of donor vectors pBGV, pBGW and pBDR that confer expression of mCFP, mCitrine and tdTomato respectively, when they are correctly inserted. The injection mix containing the three donor vectors along with iCre and PhiC31o mRNAs was microinjected into the fertilized eggs harboring hemizygous targeted allele (Figure 4A). The injected eggs were cultured up to blastocyst stage and observed for transgene expression under a fluorescent microscope (Figure 4C). Among a total of 207 eggs injected in two experimental days (89 + 118, Experiment 11 and 14 in Additional file 1: Table S2), 37 eggs developed to blastocysts and 8 of them showed either green (5 embryos) or red fluorescence (3 embryos) (Figure 4C and D) and the remaining 29 embryos were non-fluorescent. Nested PCR assay revealed successfully targeted insertion of DOI in all the 8 blastocysts (Figure 4B and C) with targeted insertion efficiency of 21.6% injected zygotes that developed into blastocysts (3.9% of total injected zygotes) (Figure 4D).

Next, we tested whether multiple Tg fetuses or mice can be obtained simultaneously by co-injecting two or three different donor vectors along with iCre and PhiC31o mRNAs into zygotes and transferring them to pseudopregnant recipients (Figure 4A). The microinjections were repeated on three separate sessions. As shown in Figure 4C and E, it was possible to obtain simultaneously Tg lines derived from donor vectors containing two to three separate DOI. Targeted insertion efficiency was 25.0% and 17.6% or 28.6% when analyzed at newborn and fetal stages respectively. The overall targeted Tg rate ranged from 1.8 to 2.2% for injected zygotes analyzed at both the stages (Figure 4E). Taken together, the results showed that *i-*PITT enables simultaneous production of up to three targeted Tg lines with different DNA of interest.

### Recombination outcomes and genotyping

**Figure 5:**
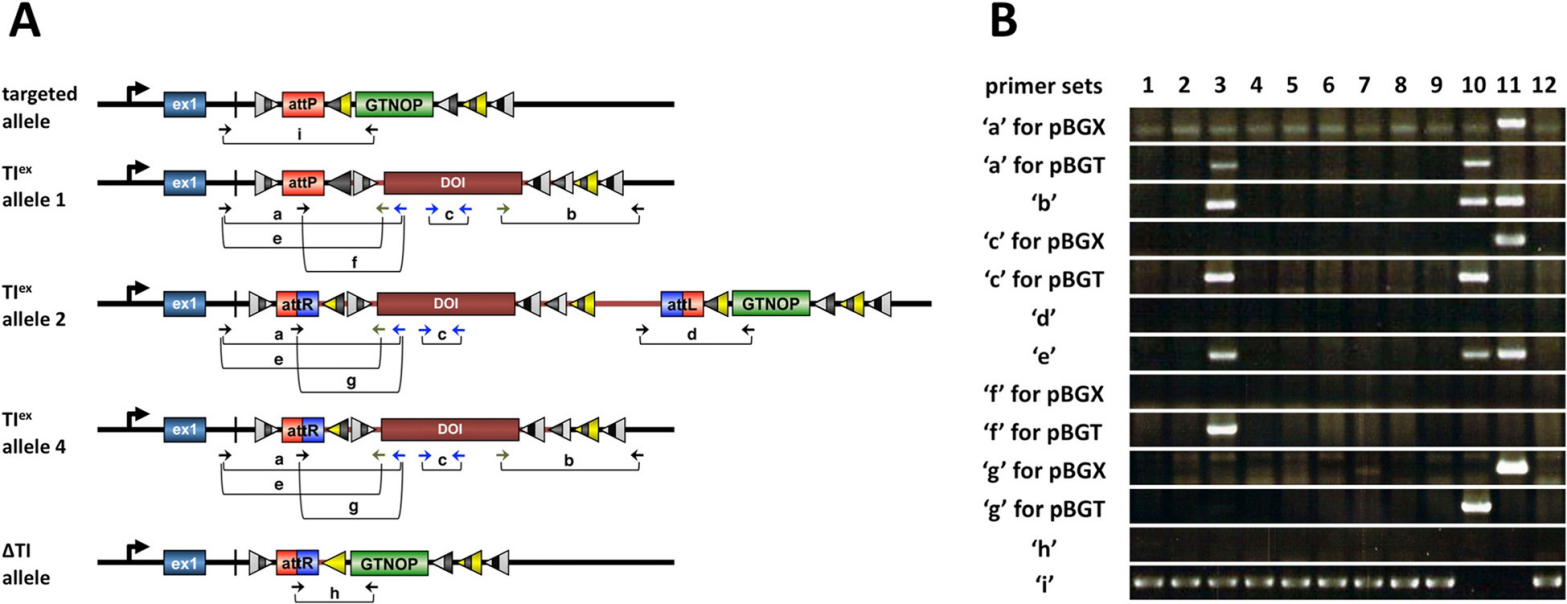
**PCR-based genotyping. (A)** The various possible allele outcomes and primer sets (a - i) used for detecting them. The black and blue arrows indicate universal and DOI-specific primers, respectively. The green arrows indicate semi-universal primers used for identification of targeted insertion for several constructs including pBGX and pBGT. **(B)** Example of PCR-based genotyping. PCR was performed using 12 samples derived from mixed injection of pBGX and pBGT (see Figure 4E). The primer sets used were as follows (from the top to the bottom): “M273/M839”, “M273/M874”, “M274/M376”, “M645/M646”, “M873/M874”, “#235/M026”, “M273/M879”, “M958/M839”, “M958/M874”, “M953/M839”, “M953/M874”, “M953/M026” and “M273/M026”. See text for more details.

Theoretically, co-introduction of both iCre and PhiC31o mRNAs can result in one of several possible insertion alleles such as TI^ex^ allele 1, 2, 4, 5 and 6 (Figure 1, Additional file 1: Figure S3 and S4). TI^ex^ allele 1 and 2 are the result of Cre-based recombination or PhiC31-based integration systems, respectively. Various genotyping assays were employed to detect targeted insertion of DOI: primer sets ‘a’, ‘b’, ‘d’ and ‘e’ amplify the 5’ or 3’ junction regions generated by recombination/integration whereas primer set ‘c’ amplifies internal regions of the transgene (Figure 5A). Targeted insertion of DOI generated by Cre-based recombination only (TI^ex^ allele 1) or by PhiC31 integration system only (TI^ex^ allele 2) can be detected with the primer sets ‘a’ and ‘b’ (for Cre-mediated recombination) or primer sets ‘a’ and ‘d’ (for PhiC31-mediated integration). Since PCR with primer set ‘a’ is applicable to identification of targeted insertion of DOI performed by either systems, we used this primer set for the first screening in all *i-*PITT experiments.

There are other possible scenarios of Cre and/or PhiC31 mediated insertions. 1) Combined effect of recombination and integration systems can generate another allele (TI^ex^ allele 4) as a result of two alternative sequential events: a) PhiC31 mediated-insertion first followed by Cre mediated-recombination via *lox2272* (Additional file 1: Figure S3), and b) Cre mediated-insertion first via *lox2272* followed by PhiC31 mediated-recombination (Additional file 1: Figure S4B). This allele can be distinguished from other alleles by PCR using primer sets ‘b’, ‘g’ and ‘f (as negative)’ (Figure 5A) or by sequencing of the PCR-amplified fragments. 2) Another possibility is that the inserted DNA may get deleted to generate ΔTI allele after two sequential events: a) PhiC31 mediated-insertion first followed by Cre mediated-recombination via *JT15/JTZ17* (Additional file 1: Figure S3), and b) Cre mediated-insertion first via *JT15/JTZ17* followed by PhiC31 mediated-recombination (Additional file 1: Figure S4A). This allele can be detected by PCR using primer set ‘h’ (Figure 5A).

PCR-genotyping of genomic DNA from *i-*PITT derived samples demonstrated that all of the positive F0 fetuses/pups harbored either TI^ex^ allele 1 or 4 (Additional file 1: Table S2; see offspring No.3 for allele 1 and offspring No.10 and 11 for allele 4 in Figure 5B). None of positive F0 fetuses/pups had TI^ex^ allele 2 (Additional file 1: Table S2), suggesting that the allele may have been changed to be TI^ex^ allele 4 immediately after Cre-mediated recombination via *lox2272* (Additional file 1: Figure S3). We also detected ΔTI allele by PCR using primer set ‘h’ (2.4% [4/169], Additional file 1: Table S2). In addition, we performed PCR with primer set ‘i’ for all the samples showing targeted insertion to detect transgene mosaicism with respect to the presence of targeted allele (Figure 5A). As a result, we could detect the transgene mosaicism in 7 out of 20 (35%) of F0 fetuses/pups (Additional file 1: Table S2; offspring No.3 individual in Figure 5B). The results shown in Figure 5B indicated that offspring No.3, 10 have a targeted fragment derived from pBGT, and offspring No.11 from pBGX. Offspring No 3 exhibited transgene mosaicism harboring TI^ex^ allele 1, while offspring No.10 and 11 were pure Tg individuals harboring TI^ex^ allele 4.

### Reproducibility of *i-*PITT technology

We next evaluated if the newly developed seed mouse can be efficiently used in another laboratory to successfully perform *i-*PITT. To test this idea, we suspended four cauda epididymides dissected from two homozygous seed mice in a 1.2 ml cryotube containing mineral oil, and shipped the cryotube to RIKEN BioResource Center (BRC) (Tsukuba, Japan) overnight under refrigerated temperatures (Additional file 1: Figure S5A), as described by Mochida *et al.* [12]. At the recipient laboratory, the epididymal spermatozoa were recovered from the transported samples and used for IVF with C57BL/6N-derived oocytes (Additional file 1: Figure S5B) and the resulting zygotes were subjected to *i-*PITT. The injection mix used in the recipient lab was from an experiment conducted at the originating lab (Figure 4E and Experiment 16 in Additional file 1: Table S2) that was shipped under frozen conditions. Among a total of 170 eggs injected 44 fetuses were recovered and 4 of them were identified as targeted Tg fetuses with a target insertion efficiency was 9.1% for fetuses recovered and 2.4% for total zygotes injected (Additional file 1: Figure S5C). This result demonstrates the robustness and reproducibility of the *i-*PITT method. Notably, because of this capability of *i-*PITT technology, there is no necessity of 1) transportation of seed mice 2) quarantine housing of the mice at the recipient facility, 3) re-derivation (cleaning process) of mice, as may be necessary in some cases, 4) maintenance of seed mice at the recipient animal facility. This system also enables that multiple different facilities can obtain the cauda epididymide samples from one central facility.

## Discussion

In this study, we designed and generated a new PITT seed mouse called TOKMO-3 to develop an *i*mproved PITT (*i-*PITT) system. The landing pad in the new seed mouse constitutes mutant *lox*P, *attP* and mutant *FRT* sites to enable targeted insertion of transgenes using Cre or PhiC31 or FLP-based PITT. The seed mouse line is with the C57BL/6N genetic background and thus the PITT derived Tg founders under pure genetic background are immediately available for analysis without the need for backcrossing. Combinatorial use of Cre-*lox*P and PhiC31-*attP/B* system resulted in improved targeted insertion efficiency of transgene from the newly designed donor vectors. The *i-*PITT strategy also enabled simultaneous production of multiple different Tg mouse lines in a single micro-injection session. The reproducibility of *i-*PITT was proven by performing it in two different laboratories.

In ES cell-based targeted insertion experiments, we confirmed that both Cre-*lox*P and PhiC31 systems worked as expected. The insertion efficiency in both Cre-*lox*P and PhiC31 systems was comparable or slightly higher in the latter than the former. To our knowledge, PITT using FLP-*FRT* system has not been developed yet. There are a few reported mutant *FRT* sites tested for recombinase-mediated cassette exchange (RMCE) in cultured cells [9,13,14]; of these, we included the most newly described genuine 48-bp variant *FRT* sites in our system. The targeted recombination efficiency of FLP-*FRT* system using mutant *FRT* sites (*F14* and *F15*) was considerably low when compared to Cre and PhiC31-based insertion. Notably, Turan et al. [9] who developed *F14* and *F15* demonstrated that RMCE employing these mutant *FRT* sites worked well when transient assay was performed using NIH3T3 cells. This implies that targeted insertion by RMCE with this *FRT* combination is possible. We found in this study that the FLP-*FRT* system failed to cause efficient targeted insertion of DOI (Figure 2A). This may partly be attributed to the ES cells used in this study and/or poor transfection efficiency of vectors in those cells. It remains unclear whether the FLP-*FRT* system can yield good *in vivo* insertion efficiency of DOI. Nevertheless, the advantages of FLP-*FRT* system in the *i-*PITT are, i) it allows removal of extra sequences for generation of the “clean” inserted allele (TI^Δex^ allele) and ii) combinatorial use of FLP-*FRT* and Cre-*lox*P systems would result in increased targeted insertion frequency of DOI, as described below (Figure 2B).

The combinatorial use of Cre-*lox*P and PhiC31 systems resulted in an increased targeted insertion efficiency in ES cells and in zygotes. While it remains unclear why simultaneous introduction of the two systems leads to increased insertion efficiency of DOI, a few possible scenarios and plausible reasons are discussed here. 1) Cre-*lox*P- and PhiC31-based insertions occur independently and the increased targeted insertion efficiency could be due to a simple additive effect. 2) Cre-*lox*P- and PhiC31-based systems work cooperatively. First insertion might occur through *lox2272* in Cre-*lox*P-based system that generates transient unstable allele (TI^ex^ allele 6; Additional file 1: Figure S4B) which can be subsequently converted to either ‘TI^ex^ allele 1’ through *JT15*/*JTZ17* recombination or ‘targeted allele’ through *lox2272* by further Cre activity. Alternatively, the unstable TI^ex^ allele 6 produced by initial Cre action may be used as a substrate for PhiC31 action to convert the allele to TI^ex^ allele 4 (Additional file 1: Figure S4B), which may add synergistic effect to increase the insertion efficiency. Such a cooperative effect was also observed when Cre-*lox*P and FLP-*FRT* systems are employed together as shown in Figure 2B. It should be noted that the TI^ex^ allele 4 can be generated not only through “Cre first-PhiC31 next” recombination/integration event (see Additional file 1: Figure S4B), but also through “PhiC31 first-Cre next” event (see Additional file 1: Figure S3). The present co-injection strategy results in generation of only TI^ex^ allele 1 or 4 (but not TI^ex^ allele 2). This presents another advantage of this system, since nearly “clean” inserted allele lacking the vector can be obtained. On the other hand, our current donor vector design has a risk that self-elimination of inserted cassette occurs, leading to generation of ΔTI allele (see Additional file 1: Figures S3 and S4A). In fact, we observed the presence of ΔTI allele in 2.4% of F0 mice (see Additional file 1: Table S2). Such unwanted outcomes can be avoided by reversing the order of *attB* and *JTZ17* in the donor vector, i.e., from “*attB*-*JTZ17*” to “*JTZ17*-*attB*”. Thus, we presume that *JTZ17*-*attB* architecture in the donor vector and the employment of tandemly arranged six copies of *JTZ17* (6x*JTZ17*) can further improve insertion efficiency of DOI to make the *i-*PITT system even more efficient.

Although complementary, the *i*-PITT system provides advantages to the current state of use of newer nuclease-mediated genome editing technologies, such as CRISPR/Cas9, for targeted insertion [15,16]. For example, 1) compared to the use of the well-tested and reliably-used recombinase systems, additional development and testing of CRISPR/Cas9 to generate multiple genetic insertion events will be necessary to ensure widespread use and application. 2) The designer nucleases can also introduce un-wanted alleles such as indels along with targeted insertion allele in a mosaic fashion, which often makes it challenging to identify the desired mutation by genotyping. On the other hand, *i*-PITT system generates only the expected target insertion allele that can be easily identified by genotyping. 3) Designer nuclease strategies often require re-constructing of targeting vectors with significantly longer homology arms that would occasionally force researchers to compromise on the size of the inserted cassettes. On the contrary, the PITT donor vectors readily accept cassettes as high as 10 kb size: these vectors do not require re-designing and building of special targeting vectors with homology arms for each project.

Simultaneous injection of multiple donors in separate sessions resulted in production of as many as three distinctly targeted Tg mice, each of which contained a transgene with a single-copy configuration. Notably, the number of zygotes used was well under 200 in each session, while traditional approaches typically use more than 200 zygotes to generate Tg founders for only one Tg construct, *i*-PITT offers over three fold efficiency in generating Tg founders for multiple transgenes. Thus, the *i*-PITT strategy could serve as a high-throughput and cost-saving method to produce targeted Tg mice. This is in contrast with the traditional production of Tg mice which is based on random insertion of multiple copy numbers of transgenes [2]. Although traditional transgenesis-based pronuclear injection of DNA containing multiple transgenes has been used for production of double or triple Tg mice that contain multiple transgenes per animal [17-19], such a strategy is not commonly followed due to inherent and obvious problems that come along with it. Another major pitfall of such multiple transgene injection strategies using traditional approaches is that several lines need to be screened to obtain reliable Tg strains for further work.

The mouse strain C57BL/6N has been considered as one of the most widely and frequently used strains in many fields of research [8,20]. Despite the usefulness of this inbred strain, many random insertion-based Tg lines are still being generated using mice with mixed genetic background (e.g. B6D2F2 and B6SJLF2) or FVB/N inbred strain because the Tg rate is more efficient and less difficult with hybrid or FVB/N strains than with C57BL/6 (e.g. 3.0% for FVB/N, 2.1% for B6D2F1 and 1.2% for C57BL/6 [% per injected]) [21]. In this study, using the newly developed *i*-PITT system in C57BL/6 background, our Tg production efficiencies ranged from 1.7 to 19.5%. Unlike the Tg mice generated using traditional approach, the models generated using TOKMO-3 do not need to go through extensive and time-consuming steps of backcrossing. This is particularly useful when the genetic backgrounds of multiple mutants need to be maintained in a pure background. For example, tissue-specific Cre and reporter alleles can be created using TOKMO-3 lines in the correct genetic background as needed for breeding them with lines produced through global programs such as the International Knockout Mouse Consortium (IKMC) [8]. Studies have shown that tissue-specific promoter driven transgenes targeted to specific sites in the genome are reliably expressed [22-26]. The usefulness of TOKMO-3 seed mice for generation of tissue-specific promoter driven transgenes is now under investigation and has already been verified for some promoters using TOKMO-1 model, the first generation seed mouse (data not shown).

For a newly established technology to become widely usable it should be easily reproducible in other labs and be flexible. We confirmed the reproducibility and flexibility of *i*-PITT with the TOKMO-3 mice by performing it in two different facilities (by different micro-injection technicians) (see Additional file 1: Figure S5). The flexibility of the *i*-PITT to use sperm isolation and IVF to generate zygotes, instead of maintaining a stud male colony for mating with females, was also assessed. Overnight refrigerated transportation of cauda epididymides dissected from TOKMO-3 mice [12] were used in an IVF session with C57BL/6N-derived oocytes at the receiving end and found to work well. This strategy relieves the requirement to maintain TOKMO-3 mice colony at the receiving facility, and thus is time- and cost-saving and convenient. This inter-lab./institution transportation of refrigerated cauda epididymides has additional merits over the existing methods of transfer of frozen embryos/sperm or live individuals [27]. For example, the cost of transportation is significantly reduced. In addition, avoiding transportation of live mice is preferable from an animal welfare point of view [12].

The targeted transgenesis approach using *i*-PITT enables stable, reliable, predictable, and repeatable transgene expression which cannot be achieved using traditional random-insertion transgenesis approaches. The *i*-PITT technology described here can generate multiple targeted Tg models in one-step. The TOKMO-3 mouse and vectors developed in this study will be made available to the scientific community globally through Experimental Animal Division of RIKEN BRC (C57BL/6-Gt(ROSA)26Sor<tm10(PITT)Maoh>, ID: RBRC06517), a part of Federation of International Mouse Resources- (http://www.fimre.org/) and also through Tokai University (TOKMO-3 mouse). The plasmid vectors developed in this study are deposited to Addgene.org. While the *i*-PITT can be used for generating any type of transgenic mice, this technology will be particularly suitable for simultaneously generating Tg lines for multiple different polymorphic variants or mutant forms of a gene. Considering that individual Tg lines generated using traditional approaches invariably differ from each other due to the position effect variation, for the projects that require generation of multiple different transgenes and their comparison with each other, the *i*-PITT system offers the perfect solution for such high-throughput transgene production projects.

## Conclusions

Due to the inherent pitfalls that come with mouse random transgenesis methods, some groups have been developing newer methods to achieve targeted transgenesis particularly through direct injection of DNA into zygotes. The new method we report here, termed *i*mproved-PITT (*i-*PITT), offers many new features compared to the published targeted transgenesis approaches, including the original PITT. The transgenic efficiency with *i*-PITT was greatly improved with a typical range of 10-30%, up to 62%. *i-*PITT also offers the advantage of creating multiple Tg models in a single experimental session and thus serves as an analogous tool for multiplexing in Tg technology similar to CRISPR/Cas9 tool for KO technology. In summary, *i*-PITT offers i) superior targeting efficiency, ii) multiplexing capability to insert different cassettes simultaneously, iii) reduction in the number of animals used; an important animal welfare consideration, iv) uniform genetic background. Moreover, these mice will be made readily available to the scientific community.

## Methods

### Plasmid construction

iCre-, PhiC31o- and FLPo-expression plasmids (pAYC, pBCW and pBCV, respectively) were generated by cloning the PCR-amplified fragment derived from pBOB-CAG-iCRE-SD (12336, Addgene, Cambridge MA) [28], pPGKPhiC31obpA (Addgene 13795), and pPGKFLPobpA (Addgene 13793) [29] into the pCAGGS vector [30], respectively.

The targeting constructs (pBDT and pBDU) were generated by modifying the bacterial artificial chromosome (BAC) clone (RP23-324O18 derived from C57BL/6J) using recombineering method with the plasmids, pRedET (Gene Bridges GmbH, Heidelberg Germany) [31]. The kanamycin resistant gene, derived from PCR-amplified fragment from pPGK-neo-*lox*P (Gene Bridges GmbH) with primer set, “M753 and M754 (Additional file 1: Table S3)”, was inserted into the BAC clone by recombineering. The resulting modified BAC was then used for retrieving with pAEF-derived retrieval vectors (pBDD and pBDE), in which the homology regions were PCR-amplified from the BAC clone or derived from previously used plasmids (pAHU [HR4 for *Rosa26*] and pAHJ [HR3 for *Rosa26*]) constructed previously by us [6,32]. The resultant vectors were pBDP and pBDF. The fragment containing the splicing acceptor, *F14*, *attP*, *JT15*, eGFP, T2A, neomycin resistant gene, partial human OCT4 gene, polyA, *FRT-L*, *lox2272* and *F15*, of which a part was synthesized by GenScript (Piscataway, NJ) was generated and cloned into pBDP and pBDF, from which the kanamycin resistant gene had been removed. The resulting plasmids are designated as pBDT and pBDU, which had been derived from pBDP and pBDF, respectively. Both the two plasmids harbor the same long (4732-bp) homology arms (corresponding to the 3’ region downstream of the *Xba*I site of intron 1 of *Rosa26* gene), but pBDT and pBDU have 1081-bp and 1898-bp short homology arms (corresponding to the 5’ region upstream of the *Xba*I site of intron 1 of *Rosa26* gene), respectively (Additional file 1: Figure S6).

Most of the donor vectors had the following components in a 5’ to 3’ direction: which include *attB*, *JTZ17*, *F14*, DOI, *F15*, *FRT-R*, *lox2272* and a plasmid backbone (pIDTSmart Kan), a part of which had been synthesized by Integrated DNA Technologies (IDT), Inc. (Coraville, IA). The donor vectors pBFB and pBEZ had tandemly repeated 6 × *JTZ17* (for pBFB) and 6 × *attB* (for pBEZ) synthesized by TaKaRa (Kyoto, Japan) in addition to *F14*, tdTomato-pA, *F15*, *FRT-R*, and *lox2272*. In the DOI region, the following cassettes were included in each donor vector: namely, ‘tdTomato - pA - pA - hygro (hygromycin B resistance gene) - mouse phosphoglycerate kinase (PGK) promoter’ for pBER, ‘mCitrine - pA’ for pBGW, ‘tdTomato - pA’ for pBDR, ‘mCFP - pA’ for pBGV, ‘Dre (derived from custom codon-optimized Dre synthesized by TaKaRa) - pA’ for pBGX, ‘iRFP - pA’ for pBGT, and ‘hamster PrP (prion protein) promoter - rabbit β-globin intron - mouse ferritin heavy chain 1 (mFTH1) - pA’ for pBGO [33-37].

The nucleotide sequences of junctional portions in the recombinant clones and PCR-amplified fragments were confirmed by sequencing. These plasmids will be available to the scientific community through Addgene. The sequences and features of recombinase/integrase recognition sites are shown in Additional file 1: Table S4.

### ES cell targeting, chimera production and establishing of seed mouse

For *in vitro* experiments, the linearized targeting vectors, pBDT and pBDU, were introduced into 129/Ola-derived E14.1 ES cells by liposomal transfection using Lipofectamin 2000 (Invitrogen, Carlsbad, CA). After selection with G418, the emerging ES cell colonies were picked and subjected to the PCR-based screening using primer sets (M024/M195 and M372/M195). The resultant ES cell clones were designated as #BDT or #BDU and one of the clones #BDU7, was used for *in vitro* study as described below. To generate TOKMO-3, the linearized pBDT was introduced into C57BL/6N-derived EGR-101 ES cells by electroporation [38]. After transfection, G418-resistant ES cell colonies were picked and subjected to the PCR-based screening using primer sets M024/M195 and M372/M195 for short arm and M275/M837 for long arm (Additional file 1: Figure S6).

The successfully targeted ES clones were then microinjected into the blastocoels of ICR blastocysts to produce chimeric mice. Germ-line transmission was confirmed for two ES cell clones (BDT#73 and BDT#78, derived from EGR-101 ES cells) by crossing the resulting male chimeric mice to C57BL/6N female mice (data not shown). Further experiments were performed using offspring of BDT#73 line and the homozygous line was established by intercrossing heterozygotes. PCR-based genotyping was performed using primers “M273, M274 and M026” (Additional file 1: Table S3), which yield 429-bp and 630-bp fragments corresponding to the wild-type allele and targeted alleles, respectively.

### Testing for targeted insertion in ES cells

ES cells (2×10^5^: #BDU7 [and E14.1 as a negative control]) were seeded onto one well of a 6-well plate, without the feeder cells. One day after seeding, transfection was performed using Lipofectamin 2000 as follows: a total of 5 μg of circular donor vector(s) containing DOI was mixed with 5 μg (or without) of circular recombinase/integrase expression plasmids pAYC (iCre), pBCW (PhiC31) and/or pBCV (FLPo) plasmids. Three days after transfection, expression of tdTomato-derived fluorescence in dissociated ES cells was analyzed using FACS LSRFortessa and FlowJo analysis software (Tree Star, Inc., Ashland, OR, USA).

### Preparation of mRNA

iCre mRNA preparation has been described previously [11]. Same batch of iCre mRNA that were used in our previous report was used. We constructed a PhiC31o mRNA expression plasmid (pBBK) by replacing the eGFP cDNA in pcDNA3.1EGFP-poly (A83) with the PhiC31o-coding sequence (derived from pPGKPhiC31obpA) [29,39]. The pBBK was linearized using *Xba*I digestion, and PhiC31o mRNA was then *in vitro* transcribed using mMESSAGE mMACHINE T7 Ultra Kit (Ambion, Austin, TX) followed by purification of mRNA using MEGAclear Kit (Ambion). To avoid clogging during microinjection, mRNAs were filtered by passing through an Ultrafree-MC filter (HV; 0.45 μm in pore size; #UFC30HV00; Millipore, Billerica, MA) before mixing them with the donor plasmids.

### Titration of PhiC31o mRNA concentration

To determine the optimal concentration of PhiC31o mRNA enabling efficient targeted transgenesis of DOI at zygote stage, we injected a solution containing various concentrations (from 0 to 45 ng/μl) of PhiC31o mRNA and 10 ng/μl of pBER donor vector into fertilized eggs carrying a heterozygous targeted allele at the *Rosa26* locus. The injected eggs were cultured in KSOM medium covered with paraffin oil up to blastocyst stage to assess their survival rate and expression of tdTomato fluorescence. The results concerning optimization of PhiC31o mRNA microinjection are compiled in Additional file 1: Table S1.

### Microinjection

Donor vector DNA and iCre and/or PhiC31o mRNAs were mixed in EmbryoMax Injection Buffer (#MR-095-10F; Millipore). Final concentration (10 ng/μl) of the donor vector was kept constant in all experiments, while the iCre and/or PhiC31o mRNA concentrations were altered, depending on the type of experiments. The DNA/mRNA mixtures were kept at −80°C until use.

Unfertilized oocytes isolated from super-ovulated female mice (C57BL/6N) were subjected to IVF with spermatozoa obtained from a homozygous TOKMO-3 male mouse. Microinjection of the DNA/mRNA mixture was performed both into pronuclei and the cytoplasm of *in vitro* fertilized eggs [11]. The injected embryos were cultured up to blastocyst stage to assess their survival rate and targeted insertion efficiency or transferred into the uteri of pseudopregnant ICR females to allow further development. The resulting fetuses (from days 13.5 to 18.5) or newborn offspring were subjected to genotyping analysis to assess successful targeted transgenesis.

### Preparation and transportation of epididymides

Procedure for inter-laboratory transportation of epididymides was performed according to the method previously described [12]. Briefly, two adult male homozygotes (TOKMO-3) were sacrificed to dissect cauda epididymides together with a part of the corpus epididymis and vas deferens. They were immediately plunged into cryotubes containing cooled mineral oil. The cryotubes were packed in a plastic bag and kept in a thermos flask containing water at 5-8°C. The samples were then transported under refrigerated condition from Tokai University (Kanagawa Prefecture, Japan) to RIKEN BioResource Center (Ibaraki Prefecture, Japan) using a commercial courier service that typically takes about 18 h for delivery. At RIKEN BioResource Center, IVF was performed using oocytes derived from super-ovulated C57BL/6N female mice (10-week-old) by incubating spermatozoa recovered from the delivered epididymides. Fertilized eggs for microinjection were obtained via IVF using the spermatozoa with 65-80% motile activity were used for this experiment.

### Detection of targeted transgene insertion

Correct insertion of donor vectors into the *Rosa26* locus was assessed by observing under a fluorescence microscope and/or PCR-based genotyping. The primer sets were designed to amplify the junctional region generated by recombination. For detection of targeted insertion of transgene at blastocysts, genomic DNA from a single blastocyst was isolated using the PicoPure DNA extraction kit (#KIT0103; Arcturus, Mountain View, CA) according to manufacturer’s instructions with the exception that an embryo was lysed in 10 μl of lysis buffer in a 0.2-mL microtube at 65°C for 3 h and 1 μl lysate was used for nested PCR. The first round of PCR reaction with the outer primer pair sets (M022/M338 for pBER and pBDR insertion, and “M022/M026” for pBGV and pBGW insertion) was performed using TaKaRa LA-Taq (#RR002A; TaKaRa) in a total of 10 μl solution. Then, 0.5 μl of the first PCR product was used as template in a nested PCR with internal primer pair sets (M273/M879) using TaKaRa Taq (#R001A; TaKaRa) with 2 × GC buffer I (#9154; TaKaRa) in a total of 10 μl solution.

For detection of targeted insertion of transgene in fetuses and newborns, genomic DNA was isolated from tail or ear piece using 50 μl of Allele-In-One Mouse Tail Direct Lysis Buffer (#ABP-PP-MT01500; Allele Biotechnology, San Diego, CA). PCR was performed in a total of 10 μl solution containing 2 × GC buffer I, 1 μl of the crude lysate and the primer pair (M273/M879 for pBER, pBDR, pBGV, pBGW, pBGX and pBGT, M274/M077 for pBER, M273/M839 for pBGX, M273/M874 for pBGT, M274/M376 for pBGX and pBGT, and M273/M955 and M274/M954 for pBGO) using TaKaRa Taq. We also used other primer sets (M273/M026 for detection of mosaic insertion, #235/M026 for detection of TI^ex^ allele 2, M953/M026 for detection of ΔTI allele, M645/M646 for Dre gene, M873/M874 for iRFP gene, M958/M839 for TI^ex^ allele 1 of pBGX, M958/M874 for TI^ex^ allele 1 for pBGT, M953/M839 for TI^ex^ allele 2 or 4 for pBGX and M953/M874 for TI^ex^ allele 2 or 4 for pBGT).

The nucleotide sequences of some PCR-amplified regions were confirmed by sequencing.

### Mice

All mice were kept in the Center of Genetic Engineering for Human Diseases (CGEHD) animal facility at Tokai University School of Medicine and the RIKEN BioResource Center. Inbred C57BL/6N and outbred ICR mice were purchased from CLEA Japan Inc. (Tokyo, Japan). The new seed mice (TOKMO-3) described here were maintained as homozygotes with an inbred genetic background of C57BL/6N. Mice are fed ad libitum under 12:12 light and dark cycle. All the animal experiments were performed in accordance with institutional guidelines and were approved by The Institutional Animal Care and Use Committee at Tokai University (Permit Number: #121007, #132013, #143037) and the RIKEN BioResource Center.

### Fluorescent microscopy of blastocysts and fetuses

Expression of fluorescence in blastocysts was inspected under an Olympus IX70 inverted fluorescence microscope (Olympus, Tokyo, Japan) with filter sets (U-MNIBA and U-MWIG). Fluorescence in fetuses was checked using the Leica M165 FC (Leica, Wetzlar, Germany) with filter sets for CFP, YFP and red fluorescence.

## Additional file

Additional file 1:
**The following additional data files that contain six figures (S1-S6) and four Tables (Table S1-S4) are available with the online version of this paper.** **Figure S1.** Schematic of targeted insertion via FLP-*FRT* system. **Figure S2.** Comparison of insertion efficiencies of iCre and PhiC31 strategies in ES cells. **Figure S3.** Schematic of possible outcomes of co-injection of iCre and PhiC31o mRNAs (“PhiC31 first-Cre next” scenario). **Figure S4.** Schematic of possible outcomes of co-injection of iCre and PhiC31o mRNAs (“Cre first-PhiC31 next” scenario). **Figure S5.** *i-*PITT at a distant laboratory using the new seed mice TOKMO-3. **Figure S6.** Schematic of targeting of *i-PITT-landing-pad* at the *Rosa26* locus. **Table S1.** contains the optimization of PhiC31o mRNA concentration data and survival rates of embryos up to blastocyst stage upon injection of PhiC31o mRNA and donor vector DNA solution. **Table S2.** contains the results of micro-injection experiments. **Table S3.** contains the primers used in the present study. **Table S4.** contains the list of recombinase/integrase recognision sites used in this study.

## Abbreviations

PI: Pronuclear injection
Tg: Transgenic
PITT: Pronuclear injection-based targeted transgenesis
*i*-PITT: *i*mproved-PITT
DOI: DNA of interest
TOKMO-3: *Tok*ai *M*asato *O*htsuka no.*3*
IKMC: International knockout mouse consortium
BRC: Bioresource center
IVF: *In vitro* fertilization
RMCE: Recombinase-mediated cassette exchange
BAC: Bacterial artificial chromosome
PGK: PhosphoGlycerate kinase
PrP: Prion protein
mFTH1: Mouse ferritin heavy chain 1
