# Supplementary material for: One-step generation of multiple transgenic mouse lines using an improved Pronuclear Injection-based Targeted Transgenesis (i-PITT)
Source: BMC Genomics. 2015 Apr 9;16(1):274. doi: 10.1186/s12864-015-1432-5 (PMC4404087; doi:10.1186/s12864-015-1432-5)
Supplement: Additional file 1: — The following additional data files that contain six figures (S1-S6) and four Tables (Table S1-S4) are available with the online version of this paper. Figure S1. Schematic of targeted insertion via FLP-FRT system. Figure S2. Comparison of insertion efficiencies of iCre and PhiC31 strategies in ES cells. Figure S3. Schematic of possible outcomes of co-injection of iCre and PhiC31o mRNAs (“PhiC31 first-Cre next” scenario). Figure S4. Schematic of possible outcomes of co-injection of iCre and PhiC31o mRNAs (“Cre first-PhiC31 next” scenario). Figure S5. i-PITT at a distant laboratory using the new seed mice TOKMO-3. Figure S6. Schematic of targeting of i-PITT-landing-pad at the Rosa26 locus. Table S1. contains the optimization of PhiC31o mRNA concentration data and survival rates of embryos up to blastocyst stage upon injection of PhiC31o mRNA and donor vector DNA solution. Table S2. contains the results of micro-injection experiments. Table S3. contains the primers used in the present study. Table S4. contains the list of recombinase/integrase recognision sites used in this study. [file 12864_2015_1432_MOESM1_ESM.pdf]

Supplementary data

Supplementary Figures

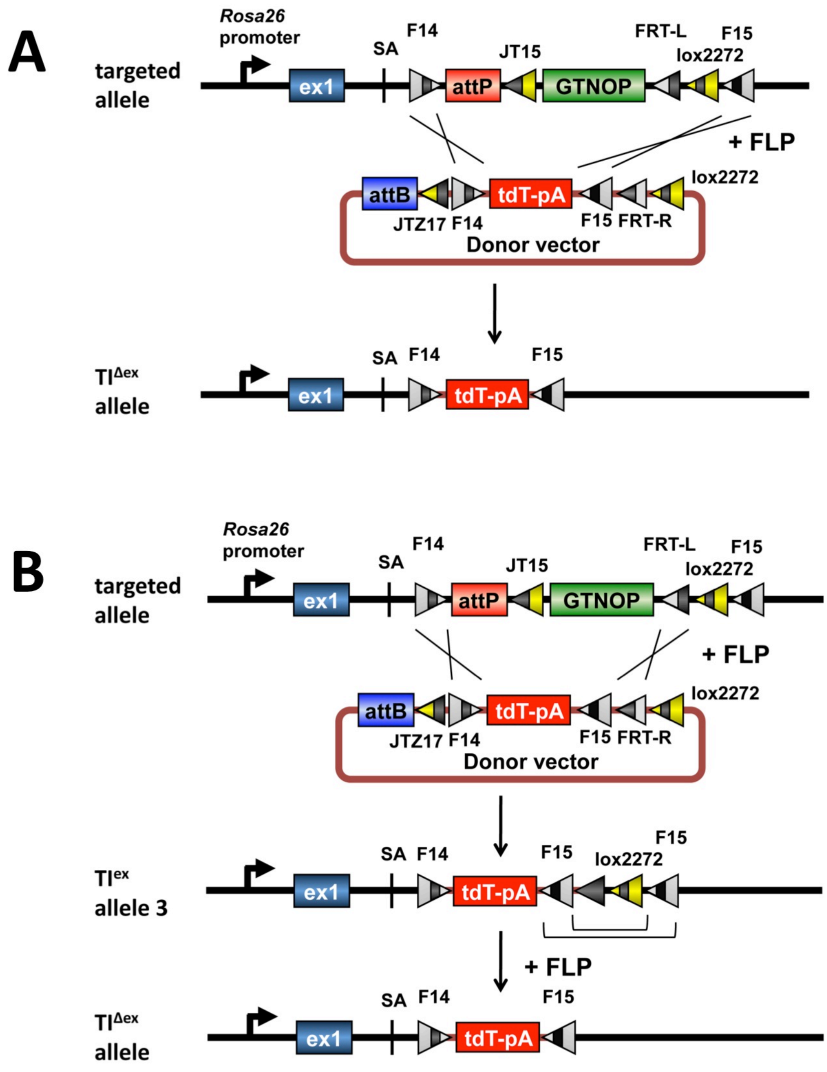

Figure S1. Targeted insertion via FLP-*FRT* system. (A) The FLP-*FRT* recombination via *F14* and *F15* results in insertion of DOI (tdTomato-pA) into the *Rosa26* locus, leading to generation of $TI^{\Delta ex}$ allele. (B) The FLP-*FRT* recombination via *F14* and *FRT-L*/*R* results in insertion of the DOI into the *Rosa26* locus, resulting in generation of TI^{ex} allele 3. Note that the recombination via *F14* and *FRT-L*/*R* requires an additional step to remove sequence between two *F15s* to obtain the final $TI^{\Delta ex}$ allele, unlike the recombination between *F14* and *F15* which directly results in $TI^{\Delta ex}$ allele (A).

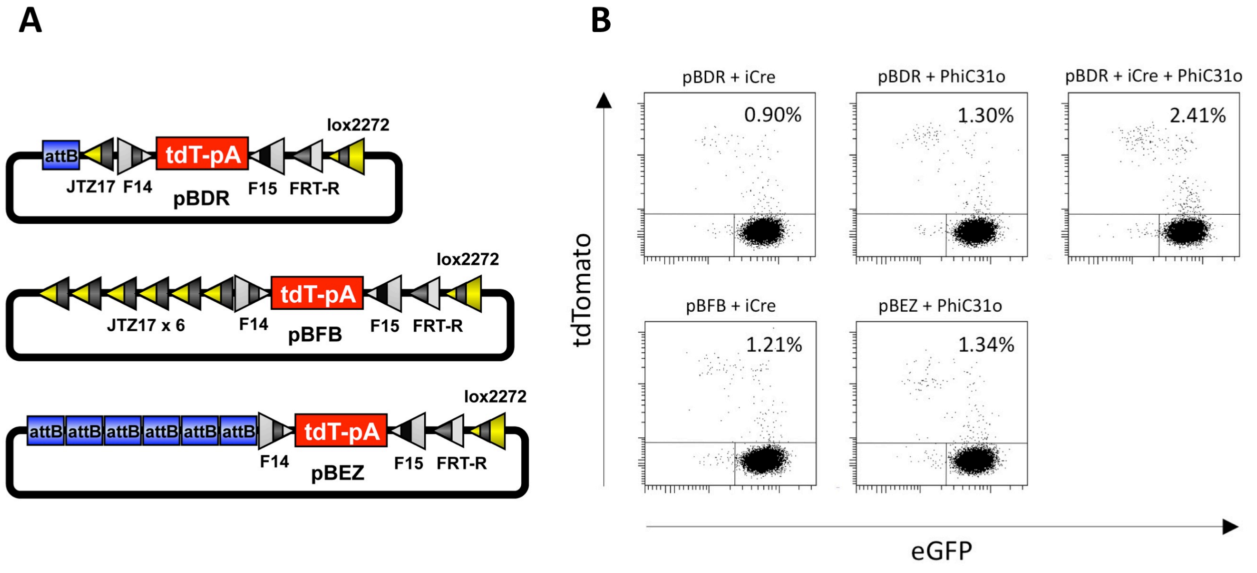

Figure S2. Comparison of insertion efficiency. (A) Structures of donor vectors used. (B) Insertion efficiency was examined by introducing the donor vector (pBDR, pBFB or pBEZ) into targeted ES cells (#BDU7) with iCre and/or PhiC31 integrase expression vector. ES cells exhibiting red fluorescence, which can be evaluated by FACS analysis, were regarded as targeted inserted clones.

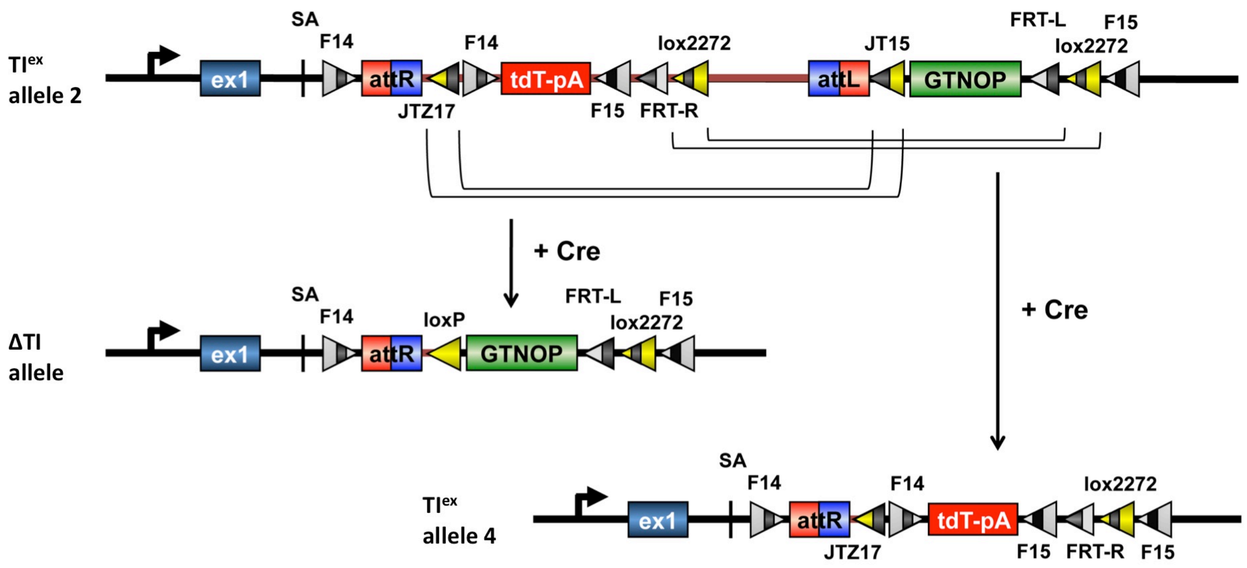

Figure S3. Possible outcomes of co-injection of iCre and PhiC31o mRNAs ("PhiC31 first-Cre next" scenario). When iCre and PhiC31o mRNAs are co-injected, TI^{ex} allele 2 generated by PhiC31 integrase will be converted into TI^{ex} allele 4 or ΔTI allele after Cre-*lox*P recombination via *lox2272* and *JT15*/*JTZ17*, respectively.

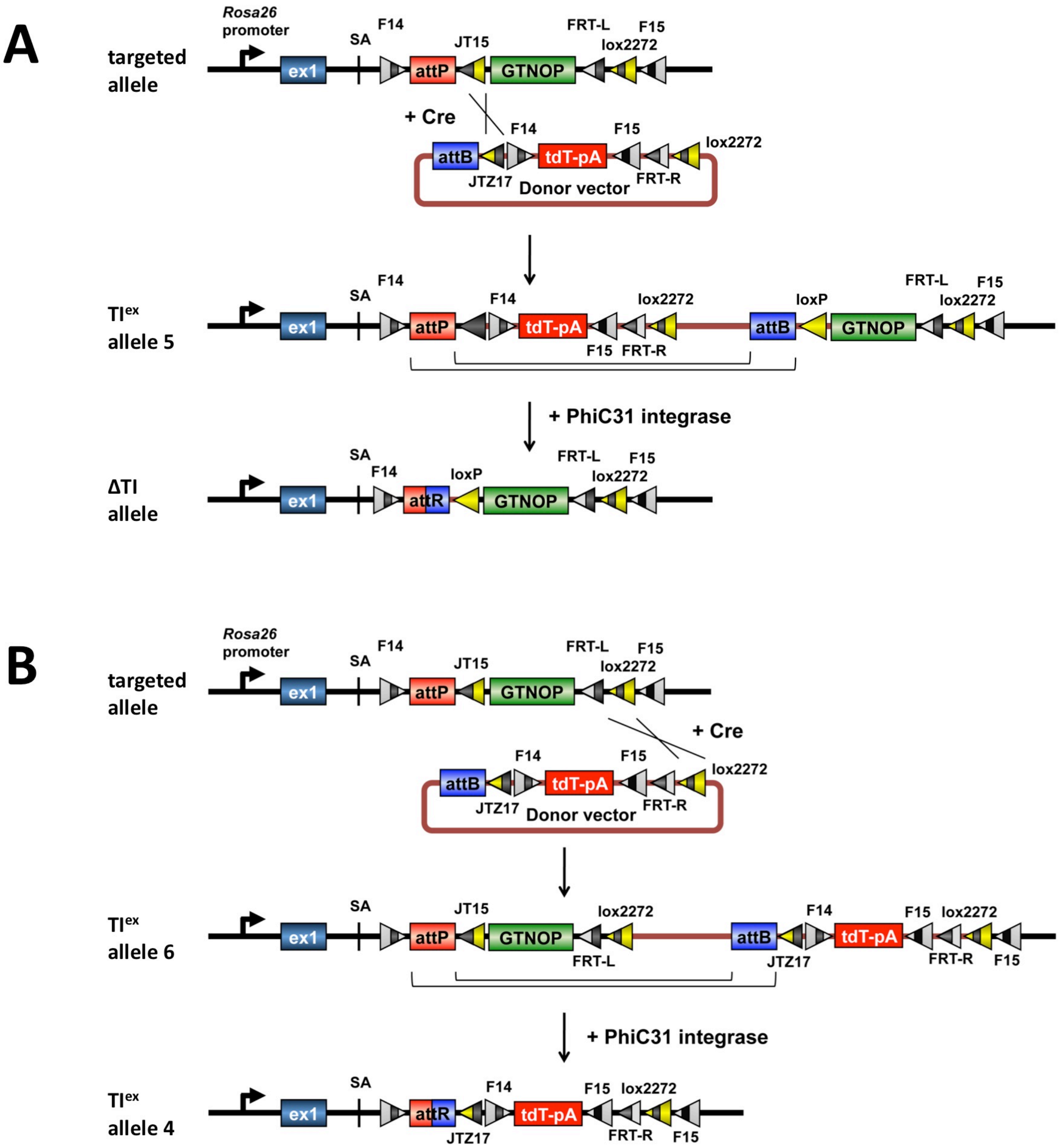

Figure S4. Possible outcomes of co-injection of iCre and PhiC31o mRNAs ("Cre first-PhiC31 next" scenario). (A) "Cre via *JT15*/*JTZ17* first-PhiC31 next" event. The Cre-*lox*P-based insertion via *JT15*/*JTZ17* generates transient allele, TI^{ex} allele 5, and then subsequent effect of PhiC31 would result in generation of ΔTI allele. (B) "Cre via *lox2272* first-PhiC31 next" event. The Cre-*lox*P-based insertion via *lox2272* generates transient allele, TI^{ex} allele 6, and then subsequent effect of PhiC31 would result in generation of TI^{ex} allele 4.

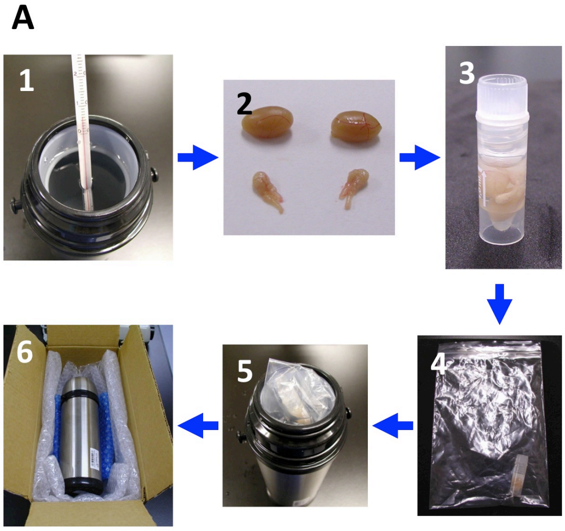

1. Thermos filled with 5-8°C water
2,3. Collect epididymides and testes from a male mouse, and put them into a cryotube containing mineral oil
4,5. Put the cryotube into a plastic bag, and immerse in cold water of thermos
6. Pack with cushions and transport at cold temperature

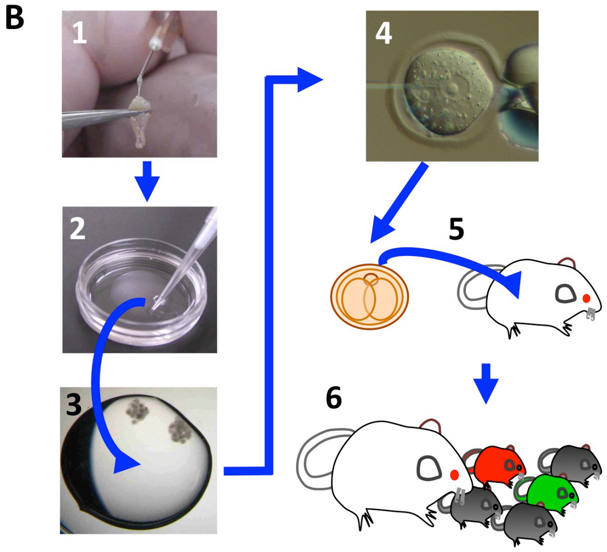

1. Pick up sperm mass
2. Preincubate for 1 h
3. Collect oocytes and fertilize them by adding sperm suspension
4. Injection of DNA/mRNA into zygotes after incubation for 5-6 h
5. Transfer 2-cell embryos into oviducts of recipients
6. Delivery of offspring

C **Trial of PITT in a second facility**

Donor Vector (size: kb)	Concentration of donor vectors (ng/μl)	Insertion system used	Concentration of mRNAs (ng/μl)	Eggs injected	Embryos transferred	Fetuses obtained (F0)	Targeted insertion (per donor vector) (% of F0; % of injected)
pBGW (3.25)<br>pBDR (3.96)	pBGW: 5<br>pBDR: 5	Cre/PhiC31	iCre: 0.5<br>PhiC31o: 7.5	170	140	44	4 (pBGW: 4, pBDR: 0)<br>(9.1%; 2.4%)

Figure S5. *i*-PITT at a distant laboratory using the new seed mice TOKMO-3. (A,B) Schematic of the procedure used in this study. The epididymides of TOKMO-3 collected at Tokai University (the first facility) were transported overnight to RIKEN BRC (the second facility) (A), and was performed in the latter facility IVF to obtain offspring (fertilized eggs) derived from TOKMO-3 (B). (C) The *i*-PITT results at the second facility.

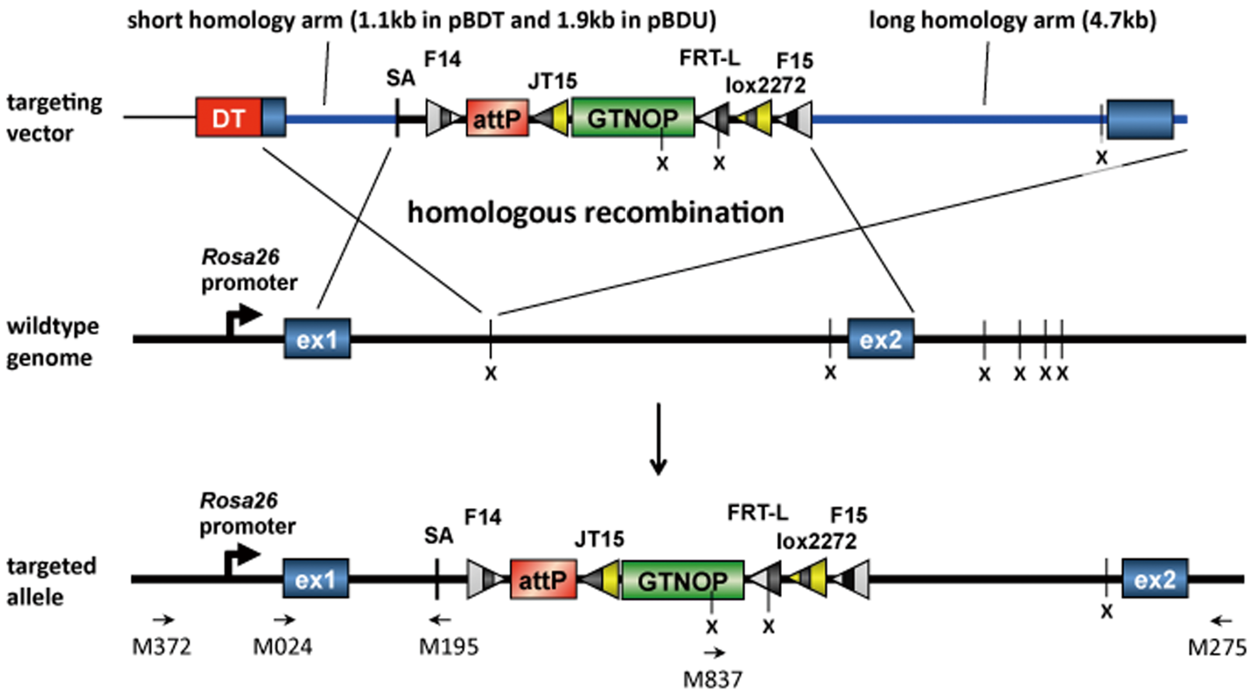

Figure S6. Schematic of targeting of *i-PITT-landing-pad* at the *Rosa26* locus. The targeting vector, pBDT (or pBDU: not shown here) comprising diphtheria toxin fragment (DT), short homology arm, splicing acceptor (SA), *F14*, *attP*, *JT15*, GTNOP (see below), *FRT-L*, *lox2272*, *F15* and long homology arm, was inserted into the *Rosa26* locus via homologous recombination in ES cells (E14.1 or EGR-101). Homology arm regions of the targeting vector are shown in blue. G418-resistant clones were picked and the targeted alleles were screened by PCR using primers shown in arrows (see details in Materials and Methods). Resultant ES cell clones, #BDU7 derived from E14.1 and BDT(#73 and #78) derived from EGR-101, were used for *in vitro* study and generation of TOKMO-3 mouse, respectively. GTNOP: a cassette containing "eGFP-T2A-Neomycin resistant gene-hOCT4-polyA". X: *Xba*I site.

Supplementary Tables

Table S1.

Table S1. Optimization of PhiC31o mRNA concentration: survival rates of embryos up to blastocyst stage upon injection of PhiC31o mRNA and donor vector DNA solution

PhiC31o mRNA concentration (ng/ul)	Eggs injected	Normal[a]	2-cell (%)[b]	4-cell (%)[b]	8-cell (%)[b]	Morula (%)[b]	Blastocyst (%)[b]	Blastocyst with red fluorescence (%)[c]
45*	23	23	11 (48)	3 (13)	3 (13)	3 (13)	5 (22)	3 (60)
22.5*	23	22	14 (64)	8 (36)	7 (32)	7 (32)	8 (36)	2 (25)
11.3*	24	23	19 (83)	15 (65)	11 (48)	11 (48)	9 (39)	2 (22)
5.6*	23	21	18 (86)	12 (57)	11 (52)	11 (52)	11 (52)	1 (9)
0*	24	23	22 (96)	21 (91)	18 (78)	18 (78)	14 (61)	0 (0)
No injection	12	-	12 (100)	12 (100)	12 (100)	12 (100)	11 (92)	0 (0)

* pBER donor vector DNA was included at 10 ng/ul concentration along with PhiC31o mRNA.

[a] Embryos with normal morphology were judged as ‘normal’ just after injection and were cultivated for further scoring. During this period, morphologic change was periodically assessed under a light microscope.

[b] The developmental ratios at each stage were assessed as (no. normal embryos developing) / (no. of normal embryos initially cultured) * 100.

[c] (Number of nornal blastocyst showing red fluorescence) / (Total number of normal blastocyst) * 100.

Table S2.

Table S2. Stepwise injection experiments.

Experiment	Donor Vector (size: kb)	concentration of donor vectors (ng/μl)	Integration system used	concentration of mRNAs (ng/μl)	Eggs injected	Developed to blastocyst (F0)	Embryos transferred	Pups/Fetuses obtained (F0)	Random integration	Targeted integration (per donor vector) (% of F0; % of injected)	Type of allele: Number	ΔTI allele/Number of F0	Number of mosaic/Number of targeted integration
1	pBER (5.99)	pBER: 10	PhiC31	PhiC31o: 15.0	25	10	-	-	ND	1 (pBER: 1)<br>(10.0%; 4.0%)	allele 2: 1	-	ND
2	pBER (5.99)	pBER: 10	Cre	iCre: 1.0	25	7	-	-	ND	2 (pBER: 2)<br>(28.6%; 8.0%)	allele 1: 2	-	ND
3	pBER (5.99)	pBER: 10	Cre/PhiC31	iCre: 0.5<br>PhiC31o: 7.5	25	9	-	-	ND	4 (pBER: 4)<br>(44.4%; 16.0%)	allele 1: 2<br>allele 2 or 4: ND	ND	ND
4	pBER (5.99)	pBER: 10	Cre/PhiC31	iCre: 1.0<br>PhiC31o: 15.0	25	16	-	-	ND	3 (pBER: 3)<br>(18.8%; 12.0%)	allele 2 or 4: 3	ND	ND
5	pBER (5.99)	pBER: 10	PhiC31	PhiC31o: 15.0	41	20	-	-	ND	4 (pBER: 4)<br>(20.0%; 9.8%)	ND	ND	ND
6	pBER (5.99)	pBER: 10	Cre	iCre: 1.0	42	14	-	-	ND	4 (pBER: 4)<br>(28.6%; 9.5%)	ND	ND	ND
7	pBER (5.99)	pBER: 10	Cre/PhiC31	iCre: 0.5<br>PhiC31o: 7.5	41	13	-	-	ND	8 (pBER: 8)<br>(61.5%; 19.5%)	ND	ND	ND
8	pBER (5.99)	pBER: 10	Cre/PhiC31	iCre: 1.0<br>PhiC31o: 15.0	45	15	-	-	ND	7 (pBER: 7)<br>(46.7%; 15.6%)	ND	ND	ND
9	pBER (5.99)	pBER: 10	Cre/PhiC31	iCre: 0.5<br>PhiC31o: 7.5	100	46	-	-	ND	13 (pBER: 13)<br>(28.3%; 13.0%)	allele 1: 9<br>allele 2 or 4: 4	ND	ND
10	pBER (5.99)	pBER: 10	Cre/PhiC31	iCre: 1.0<br>PhiC31o: 15.0	100	47	-	-	ND	13 (pBER: 13)<br>(27.7%; 13.0%)	allele 1: 5<br>allele 2 or 4: 8	ND	ND
11	pBGW (3.25)<br>pBDR (3.96)<br>pBGV (3.25)	pBGW: 3.3<br>pBDR: 3.3<br>pBGV: 3.3	Cre/PhiC31	iCre: 0.5<br>PhiC31o: 7.5	89	22	-	-	ND	5 (pBGW: 3, pBDR: 2, pBGV: 0)<br>(22.7%; 5.6%)	allele 1: 1<br>allele 2 or 4: 4	ND	ND
12	pBER (5.99)	pBER: 10	Cre/PhiC31	iCre: 0.5<br>PhiC31o: 7.5	120	-	113	28	ND	2 (pBER: 2)<br>(7.1%; 1.7%)	allele 1: 1<br>allele 4: 1	0/28	1/2
13	pBER (5.99)	pBER: 10	Cre/PhiC31	iCre: 0.5<br>PhiC31o: 7.5	122	-	114	23	ND	4 (pBER: 4)<br>(17.4%; 3.3%)	allele 1: 2<br>allele 4: 2	1/23	2/4
14	pBGW (3.25)<br>pBDR (3.96)<br>pBGV (3.25)	pBGW: 3.3<br>pBDR: 3.3<br>pBGV: 3.3	Cre/PhiC31	iCre: 0.5<br>PhiC31o: 7.5	118	15	-	-	ND	3 (pBGW: 2, pBDR: 1, pBGV: 0)<br>(20.0%; 2.5%)	allele 1: 2<br>allele 2 or 4: 1	ND	ND
15	pBGX (3.59)<br>pBGT (3.47)	pBGX: 5<br>pBGT: 5	Cre/PhiC31	iCre: 0.5<br>PhiC31o: 7.5	150	-	143	12	0	3 (pBGX: 1, pBGT: 2)<br>(25.0%; 2.0%)	allele 1: 1<br>allele 4: 2	0/12	1/3
16	pBGW (3.25)<br>pBDR (3.96)	pBGW: 5<br>pBDR: 5	Cre/PhiC31	iCre: 0.5<br>PhiC31o: 7.5	166	-	153	17	ND	3 (pBGW: 2, pBDR: 1)<br>(17.6%; 1.8%)	allele 1: 1<br>allele 4: 2	0/17	0/3
17	pBGO (5.19)	pBGO: 10	Cre/PhiC31	iCre: 0.5<br>PhiC31o: 7.5	202	-	198	31	ND	8 (pBGO: 8)<br>(25.8%; 4.0%)	allele 4: 8	1/31	3/8
18	pBGW (3.25)<br>pBDR (3.96)	pBGW: 5<br>pBDR: 5	Cre/PhiC31	iCre: 0.5<br>PhiC31o: 7.5	170	-	140	44	ND	4 (pBGW: 4, pBDR: 0)<br>(9.1%; 2.4%)	allele 4: 4	0/44	ND
19	pBGW (3.25)<br>pBDR (3.96)<br>pBGV (3.25)	pBGW: 3.3<br>pBDR: 3.3<br>pBGV: 3.3	Cre/PhiC31	iCre: 0.5<br>PhiC31o: 7.5	181	-	167	14	ND	4 (pBGW: 1, pBDR: 2, pBGV: 1)<br>(28.6%; 2.2%)	allele 4: 4	2/14	ND
Total					1787	234	1028	169		95		4/169	7/20

Table S3.

Table S3. Primers used in the present study.

#235	TTGGTATCTGCGCTCTGC
M022	TGAGCGGCTGCGGGGCGGGTGCAA
M024	CCTAAAGAAGAGGCTGTGCTTTGG
M026	GGTGGTGCAGATGAACTTCAG
M026	GGTGGTGCAGATGAACTTCAG
M077	CCATTTGTCACGTCCTGCAC
M195	CATCAAGGAAACCCTGGACTACTG
M273	TCAGTAAGGGAGCTGCAGTGG
M274	CGATGGAAAATACTCCGAGGC
M275	CACATTGCATGGATTATACAAGGTG
M338	CTCTTTGATGACGGCCATGTT
M372	GTTGCTGGTGAAGACGTTACAC
M376	TGCATTCTAGTTGTGGTTTGTCC
M645	CACCATCGACAAGCACTACG
M646	GATTCTGGCCTTGTTGCTTC
M753	CTGGGAGAATCCCTTCCCCCTCTTCCCTCGTGATCTGCAACTCCAGTCTTGATATCAAAACTCTCTTAAGGTAGCATTCTGCACGCTTCAAAAG
M754	CTGCAGGACAACGCCCACACACCAGGTTAGCCTTTAAGCCTGCCCAGAAGATATCGCTACCTTAGGACCGTTATAGTTACGAAGAACTCCAGCATGAGA
M837	TCAGCCATACCACATTTGTAGAG
M839	AGTACTCTCTGGCCATCTCG
M873	ATACCGGTCGCCACCATGGCCGAAGGGAGCGT
M874	ATGGCCGGCCTTATTCTTCCATCACGCCAATC
M879	TTGCTCACCATGGTGGCGACCGGTGA
M953	TGGGGTAACCTTTGGGCTC
M954	GGAGAGAAGGAGCCACAAAC
M955	AGCCCAAGCTTACCGGTGA
M958	TGGGGTAACCTTTGAGTTCTC

Table S4.

Table S4. List of recombinase/integrase recognision sites used in this study

recombinase/integrase recognision sites	sequence	recognized by	recombines or integrates with
attB	CCGCGGTGCGGGTGCCAGGGCGTGCCCTTGGGCTCCCCGGGCGCGTACTCCAC	PhiC31 integratse	attP
attP	GTAGTGCCCCAACTGGGGTAACCTTTGAGTTCTCTCAGTTGGGGGCGTAG	PhiC31 integratse	attB
attR	GTAGTGCCCCAACTGGGGTAACCTTTGGGCTCCCCGGGCGCGTACTCCAC	not by PhiC31 integratse without assistance	attL
attL	CCGCGGTGCGGGTGCCAGGGCGTGCCCTTGAGTTCTCTCAGTTGGGGGCGTAG	not by PhiC31 integratse without assistance	attR
JT15	AATTATTCGTATAGCATACATTATACGAAGTTAT	Cre recombinase	JTZ17
JTZ17	ATAACTTCGTATAGCATACATTATAGCAATTTAT	Cre recombinase	JT15
lox2272	ATAACTTCGTATAGGATACTTTATACGAAGTTAT	Cre recombinase	loz2272
F14	GAAGTTCCTATTCCGAAGTTCCTATTCTATCAGAAGTATAGGAACTTC	FLP recombinase	F14
F15	GAAGTTCCTATTCCGAAGTTCCTATTCTTATAGGAGTATAGGAACTTC	FLP recombinase	F15
FRT-R	GAAGTTCCTATTCCGAAGTTCCTATTCTCTAGAAAGTATATGAACTTC	FLP recombinase	FRT-L
FRT-L	GAAGTTCCTATTCCGAAGTTCATATTCTCTAGAAAGTATAGGAACTTC	FLP recombinase	FRT-R
